# MicroRNA-30a targets BECLIN-1 to inactivate autophagy and sensitizes gastrointestinal stromal tumor cells to imatinib

**DOI:** 10.1038/s41419-020-2390-7

**Published:** 2020-03-23

**Authors:** Wei Chen, Zhouqi Li, Hao Liu, Sujing Jiang, Guannan Wang, Lifeng Sun, Jun Li, Xiaochen Wang, Shaojun Yu, Jianjin Huang, Ying Dong

**Affiliations:** 1 Cancer Institute of Integrated Traditional Chinese and Western Medicine, Key Laboratory of Cancer Prevention and Therapy combining Traditional Chinese and Western Medicine, Zhejiang Academy of Traditional Chinese Medicine, 310012 Hangzhou, Zhejiang China; 2 Department of Medical Oncology, Tongde Hospital of Zhejiang Province, 310012 Hangzhou, Zhejiang China; 3 Department of Medical Oncology, The Second Affiliated Hospital of Zhejiang University School of Medicine, 310009 Hangzhou, China; 4 Department of Radiation and Medical Oncology, The First Affiliated Hospital of Wenzhou Medical University, No.2 Fuxue Lane, 325000 Wenzhou, China; 5 Department of Surgery Oncology, The Second Affiliated Hospital of Zhejiang University School of Medicine, 310009 Hangzhou, China

**Keywords:** Cell death, Autophagy

## Abstract

Gastrointestinal stromal tumors (GISTs), the most widespread type of sarcoma, contain driver gene mutations predominantly of receptor tyrosine kinase and platelet-derived growth factor receptor alpha. However, the inevitable development of resistance to imatinib (IM) cannot be fully attributed to secondary driver gene mutations. In this study, we investigated the role of microRNA-30a in sensitization of GIST cells to IM in vivo and in vitro. Higher levels of miR-30a were detected in GIST-T1 cells, which were more sensitive to IM than GIST-882 cells. IM treatment also reduced miR-30a levels, indicating the possible role of miR-30a in GIST IM resistance. Subsequently, miR-30a was confirmed to be an IM sensitizer via a mechanism that was attributed to its involvement in the regulation of cell autophagy. The interaction of miR-30a and autophagy in IM treated GIST cells was found to be linked by beclin-1. Beclin-1 knockdown increased IM sensitivity in GIST cell lines. Finally, miR-30a was confirmed to enhance IM sensitivity of GIST cells in mouse tumor models. Our study provides evidence for the possible role of miR-30a in the emergence of secondary IM resistance in GIST patients, indicating a promising target for overcoming this chemoresistance.

## Introduction

Gastrointestinal stromal tumors (GISTs) are the most common gastrointestinal mesenchymal tumors in the alimentary tract, accounting for 0.2% of all gastrointestinal tumors^1^. GISTs occur at every level of the gastrointestinal tract, but most commonly in the stomach (60–70%), and small intestine (20–30%)^2^. Receptor tyrosine kinase (*KIT*) and platelet-derived growth factor receptor alpha (*PDGFRA*) are the driver genes of GISTs and their mutations can be detected in over 85% of GIST patients^3,4^. Imatinib (IM) is a tyrosine kinase (TK) inhibitor which competitively blocks the ATP‑binding site of the TK receptor, thus inhibiting a variety of TKs, including c-Kit and PDGFRA, subsequently inhibiting signal transduction and suppressing tumor growth^5,6^. IM is now the standard first-line drug for patients with unresectable primary GISTs or metastasized recurrent GISTs^7^. Also, adjuvant therapy with IM can effectively reduce the recurrence rate after surgery, and inclusion of IM in neoadjuvant therapy is recommended to shrink tumors before surgery^8^. However, the wide use of IM in clinical cases is associated with the emergence of the secondary drug resistance^9^, limiting the effectiveness of chemotherapeutics. Thus, further investigations into the possible mechanisms responsible for IM resistance in GISTs and the identification of novel therapeutic targets to overcome this chemotherapy resistance are urgently required.

MicroRNAs (miRNAs), a class of small non-coding RNAs (18–25 nucleotides in length), regulate gene expression by binding to the 3′-untranslated region (UTR) of target genes, leading to translational repression or degradation of the targeted transcript^10,11^. Aberrant expression of miRNAs has been implicated in the pathogenesis of several diseases including cancer, and miRNAs function as either tumor suppressors or oncogenes depending on their target genes^12–14^. MicroRNA-30a (miR-30a), as a member of the miR-30 family, has similar functions to many other miRNA species and plays a role in cancer cell proliferation, invasion, metastasis, and autophagy^15^. Autophagy is a catabolic pathway that degrades and recycles cellular compartments to allow cell survival under stress conditions by orchestrating delivery of the damaged components to the lysosome^16,17^. It has been reported that autophagy is induced by IM treatment in GIST cells^18^. Moreover, depleting autophagy by silencing of autophagy regulators (ATGs) or antimalarial lysosomotropic agents sensitized GIST cells to IM^18^. Thus, we investigated the crosstalk between miR-30a and autophagy and the influence of miR-30a on IM-resistant GIST cells.

In this study, we demonstrated that miR-30a inactivates autophagy in GIST cell lines exposed to IM, and that miR-30a upregulation increases IM sensitivity of GIST cells both in vivo and in vitro. Furthermore, beclin-1 serves as the linkage between miR-30a and autophagy. Our findings elucidate the function of miR-30a during IM treatment and the underlying mechanisms, and implicating miR-30a as a promising target for overcoming IM resistance in GISTs.

## Results

### MiR-30a is associated with drug sensitivity

First, we performed qRT-PCR assays to detect the expression of a panel of miRNAs with abnormal expression in GISTs (Fig. S1A, B). Among the 15 miRNAs tested, miR-30a-5p displayed the largest decrease in expression in GIST-882 cells following IM treatment. A similar reduction in miR-30a-5p expression was observed in GIST-T1 cells after IM treatment. Moreover, we detected expression levels of the miR-30 family in GIST cells after IM treatment; we found that the expression of miR-30a was lower than that of other miR-30 family members (Fig. S1A, B). Also, we collected samples from 10 patients and analyzed the miR-30a level in GISTs and normal adjacent tissues. As we expected, RT-qPCR demonstrated that miR-30a was expressed at a much lower level in GISTs compared with normal tissues (*p* < 0.05) (Fig. S1C). Therefore, we were interested in studying miR-30a.

**Fig. 1:**
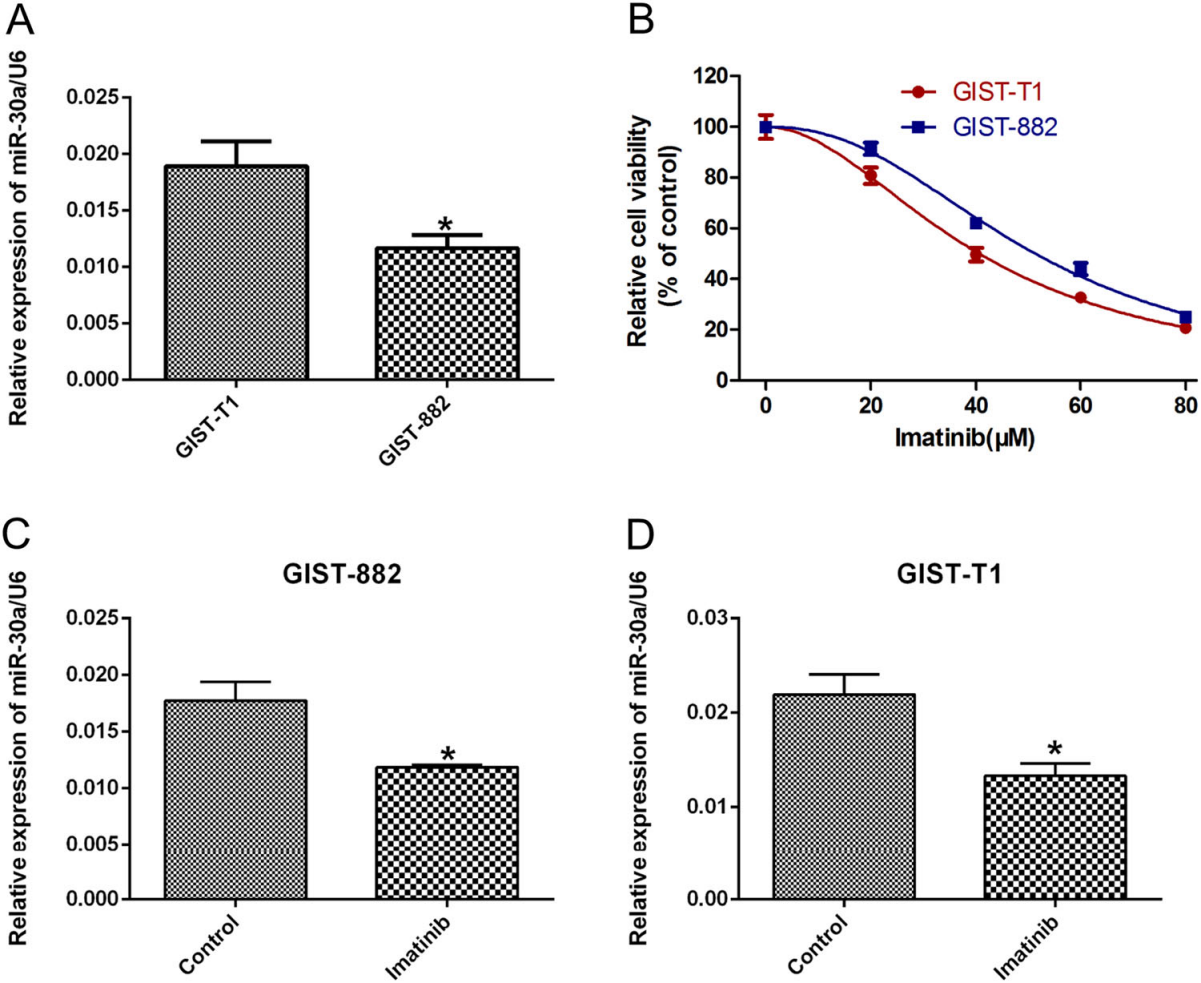
The expression levels of miR-30a are associated with imatinib sensitivity of GIST-T1 and GIST-882 cells. **a** MiR-30a was differently expressed in GIST-T1 and GIST-882 cells. *P < 0.05 vs. Control. **b** The sensitivity of GIST-T1 and GIST-882 cells to imatinib. **c**, **d** Quantitative analysis of miR-30a levels in GIST-T1 and GIST-882 cells treated with imatinib. *P < 0.05 vs. Control. All data are representative of three independent experiments.

To investigate a correlation between miR-30a and drug sensitivity, RT-qPCR assays were performed to determine the levels of miR-30a in the human GIST lines, GIST-882 and GIST-T1 cells. The basal level of miR-30a was higher in GIST-T1 cell compared to that in GST-882 cells (Fig. 1a). CCK-8 assays then showed that GIST-T1 cells were more sensitive to IM than GIST-882 cells (Fig. 1b). Moreover, miR-30a expression decreased both in GIST-882 and GIST-T1 cells after treatment with IM compared to the levels detected in the control (Fig. 1c, d). These data suggested that miR-30a is associated with GIST cell sensitivity to IM.

### MiR-30a enhances GIST cells sensitivity to imatinib

**Fig. 2:**
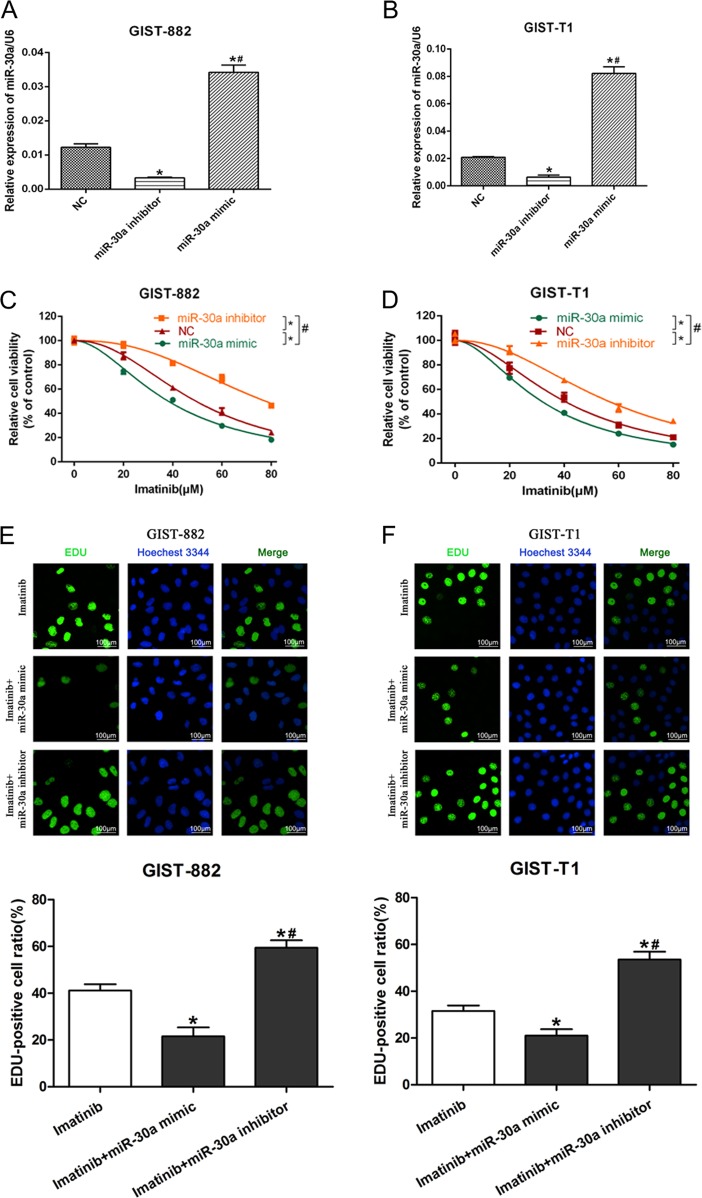
MiR-30a enhances imatinib sensitivity of GIST cells. GIST-T1 and GIST882 cells were transfected with miR-30a mimic, miR-30a inhibitor or negative controls, then cells were incubated with imatinib (IC50) for 48 h. **a**, **b** The efficiency of transfection was validated by qRT-PCR. *P < 0.05 vs. non-specific control; ^#^*P* < 0.05 vs. miR-30a inhibitor. **c**, **d** The cell viability was assessed by the CCK-8 assay. *P < 0.05 vs. non-specific control; ^#^*P* < 0.05 vs. miR-30a inhibitor. **e**, **f** The cell proliferation was measured by EdU assay. *P < 0.05 vs. NC; ^#^*P* < 0.05 vs. miR-30a inhibitor. All data are representative of three independent experiments.

To investigate the functional role of miR-30a in chemosensitivity, we transfected GIST-882 and GIST-T1 cells with either miR-30a mimic or miR-30a inhibitor. As shown in Fig. 2a, b, qRT-PCR analysis confirmed that the miR-30a level was higher in the miR-30a mimic transfected group compared with those in the NC group, and also that the miR-30a inhibitor effectively inhibited miR-30a expression. CCK-8 assays showed that transfection of miR-30a mimic significantly enhanced the cytotoxicity of IM in GIST-882 cells, whereas introduction of the miR-30a inhibitor had the opposite effects (Fig. 2c). Similar effects were observed on GIST-T1 cells (Fig. 2d). In addition, EdU analysis indicated that miR-30a overexpression significantly suppressed the proliferation of IM treated GIST-882/GIST-T1 cells. In contrast, miR-30a knockdown stimulated proliferation (Fig. 2e, f). These data demonstrated that miR-30a overexpression increased the sensitivity of cancer cells to IM treatment.

### MiR-30a participates in the regulation of cell autophagy

**Fig. 3:**
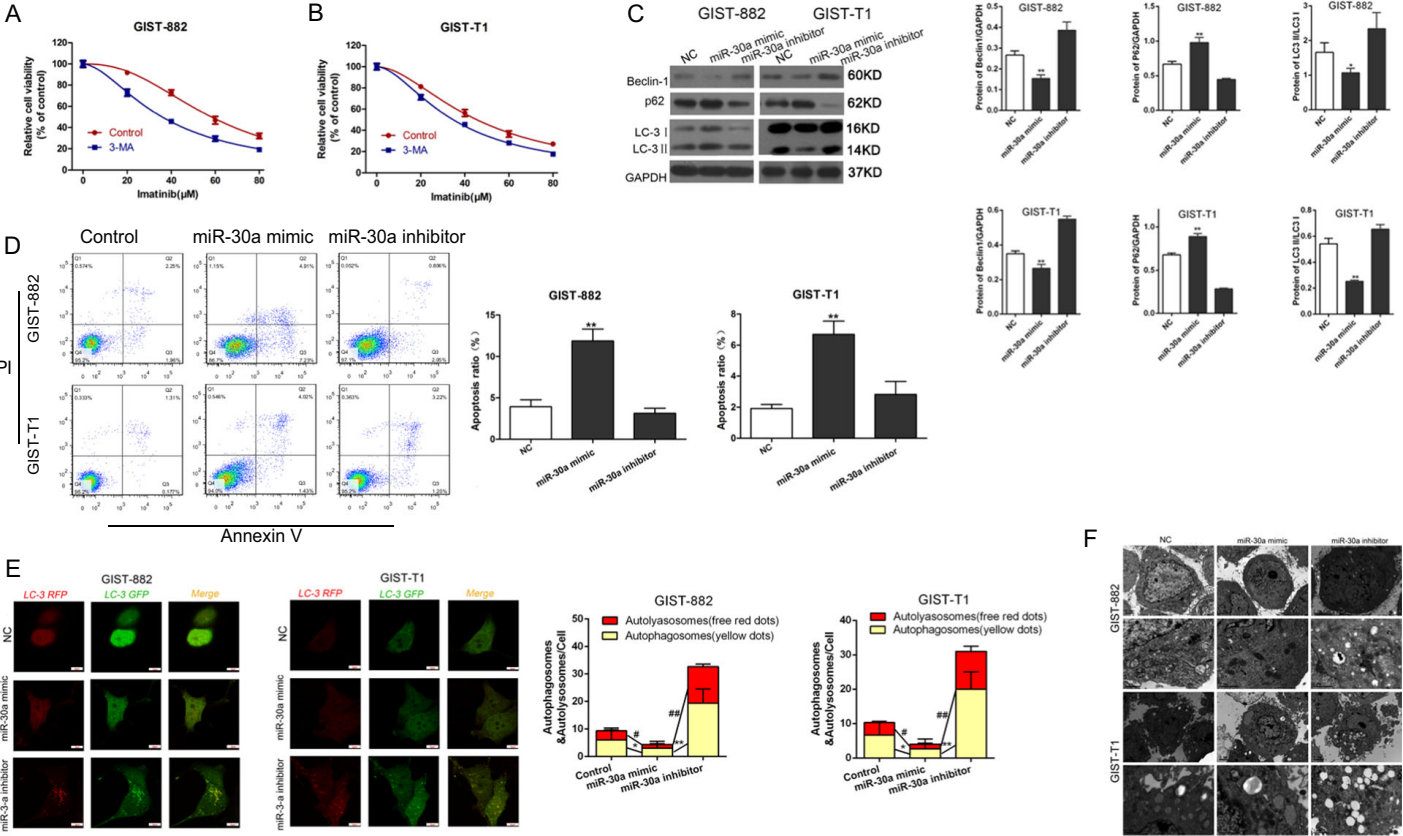
MiR-30a regulates cell autophagy and apoptosis in GIST-T1/GIST-882 cells. **a**, **b** CCK-8 assay was performed to measure the relative viability of GIST-T1 and GIST-882 cells treated with imatinib and 3-MA. **c** Flow cytometric analysis was employed to determine the effect of miR-30a on the apoptosis rates in GIST cells. *P < 0.05, **P < 0.01 vs. non-specific control. **d** Western blotting was applied to assess the levels of autophagy-related proteins. **P < 0.01 vs. non-specific control. **e** The effects of miR-30a on autophagosome formation shown by confocal laser scanning microscopy in GIST-T1/GIST-882 cells. Red dots represent autolyasosomes. Yellow dots represent autophagosomes. *P < 0.05, **P < 0.01 vs. NC. **f** Electron microscopy was used to observe autophagic vacuoles in GIST-T1/GIST-882 cells after infected with miR-30 mimics or inhibitors. All data are representative of three independent experiments.

It has been reported that miR-30a is a robust autophagy inhibitor and is responsible for reduced IM-induced apoptosis in chronic myeloid leukemia cells via an autophagy-related mechanism^19^. Thus, we hypothesized that autophagy contributes to the ability of miR-30a to influence the sensitivity of GIST cells to IM. To test this hypothesis, we showed that treatment with the autophagy inhibitor 3-methyladenine (3-MA) enhanced cell sensitivity to IM, with a greater effect observed in GIST-882 cells, which are more resistant to IM than GIST-T1 cells (Fig. 3a, b). These results suggested that cell resistance to IM is autophagy-mediated. To further investigate the effects of miR-30a on autophagy, we transfected cells with miR-30a mimic or miR-30a inhibitor and analyzed cell resistance to IM by western blotting. MiR-30a re-expression decreased autophagy-related protein expression levels in GIST cells compared with those in NC cells, while miR-30a inhibition increased the expression levels of those proteins in IM treated cells compared with those in NC cells (Fig. 3c). In addition, flow cytometric analysis indicated a clear increase in apoptosis in GIST cells following miR-30a re-expression, whereas miR-30a inhibition had the opposite effects (Fig. 3d). Besides, confocal laser scanning microscopy and electron microscopy showed that the numbers of LC3 puncta and autophagic vacuoles were significantly reduced in cells overexpressing miR-30a, while the LC3 puncta formation and autophagic vacuoles were increased in GST cells following the introduction of the miR-30a inhibitor (Fig. 3e, f). Taken together, our findings confirmed that miR-30a participates in the regulation of cell autophagy in GIST cell lines, rescuing them from apoptosis.

### MiR-30a inhibits autophagy during imatinib treatment

**Fig. 4:**
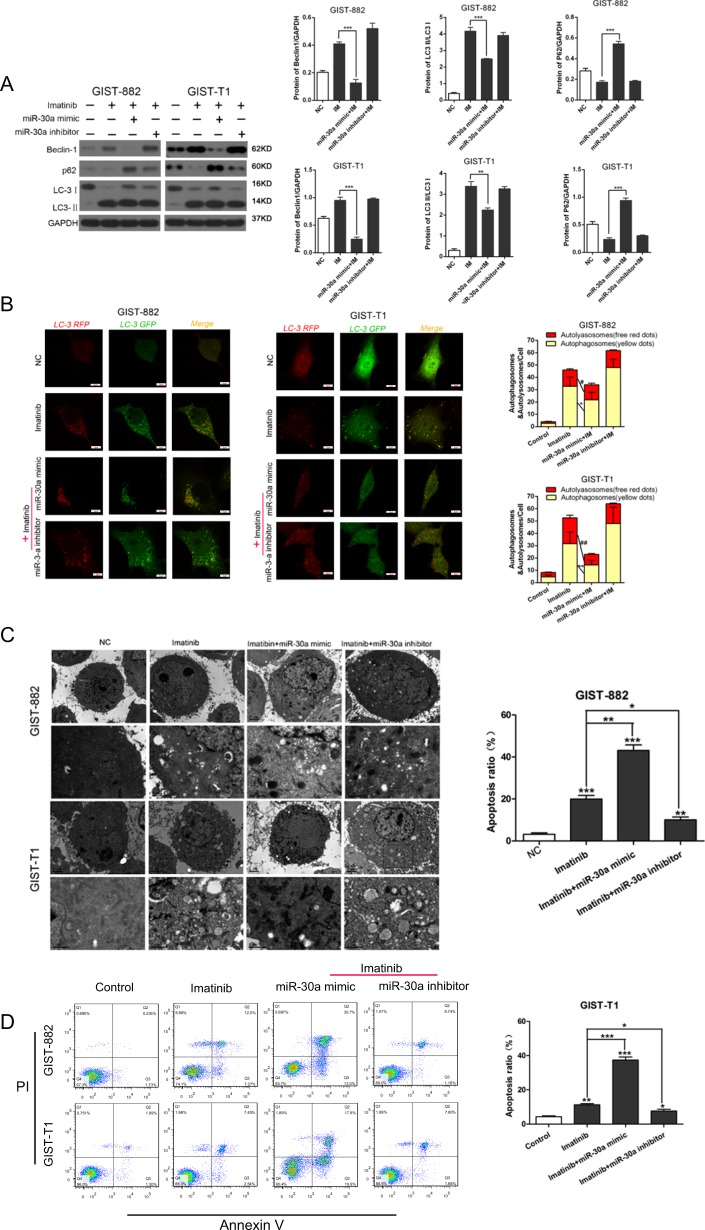
MiR-30a attenuates imatinib-induced cell autophagy and apoptosis in GIST-T1/GIST-882 cells. GIST-T1/GIST-882 cells were transfected with miR-30a mimic or miR-30a inhibitor, then cells were incubated with imatinib (IC50) for 48 h. **a** The autophagy-related proteins were measured by Western blot. **P < 0.01, ***P < 0.001 vs. IM. **b** Confocal laser scanning microscopy was used to examine autophagosome formation. Red dots represent autolyasosomes. Yellow dots represent autophagosomes. *P < 0.05, **P < 0.01 vs. IM. **c** Autophagic vacuoles were measured by electron microscopy. *P < 0.05, **P < 0.01, ***P < 0.001 vs. IM. **d** The apoptotic cell ratio was assessed by flow cytometry. *P < 0.05, **P < 0.01, ***P < 0.001 vs. IM. All data are representative of three independent experiments.

To further investigate the functional role of miR-30a in IM-induced autophagy, we exposed GIST-882/GIST-T1 cells to IM and observed increased autophagic flux, a change reflected by the enhancement of LC3-II/I expression ratio. However, miR-30a overexpression partially reversed IM-induced LC3-II accumulation in GST cells. In contrast, treatment with the miR-30a inhibitor enhanced IM-mediated autophagic flux (Fig. 4a). In accordance with the results of western blot analysis described previously, we found that the miR-30a mimic suppressed the formation of IM-mediated LC3 puncta and autophagic vacuoles compared with those detected in the IM, whereas the miR-30a inhibitor mediated the opposite effects (Fig. 4b, c). Furthermore, treatment with the miR-30a mimic increased the apoptosis ratio, while the miR-30a inhibitor decreased the ratio (Fig. 4d). These data suggested that miR-30a functions as a negative regulator of IM-mediated autophagy in GIST cell lines.

### MiR-30a sensitizes GIST cells to imatinib by inhibiting autophagy

**Fig. 5:**
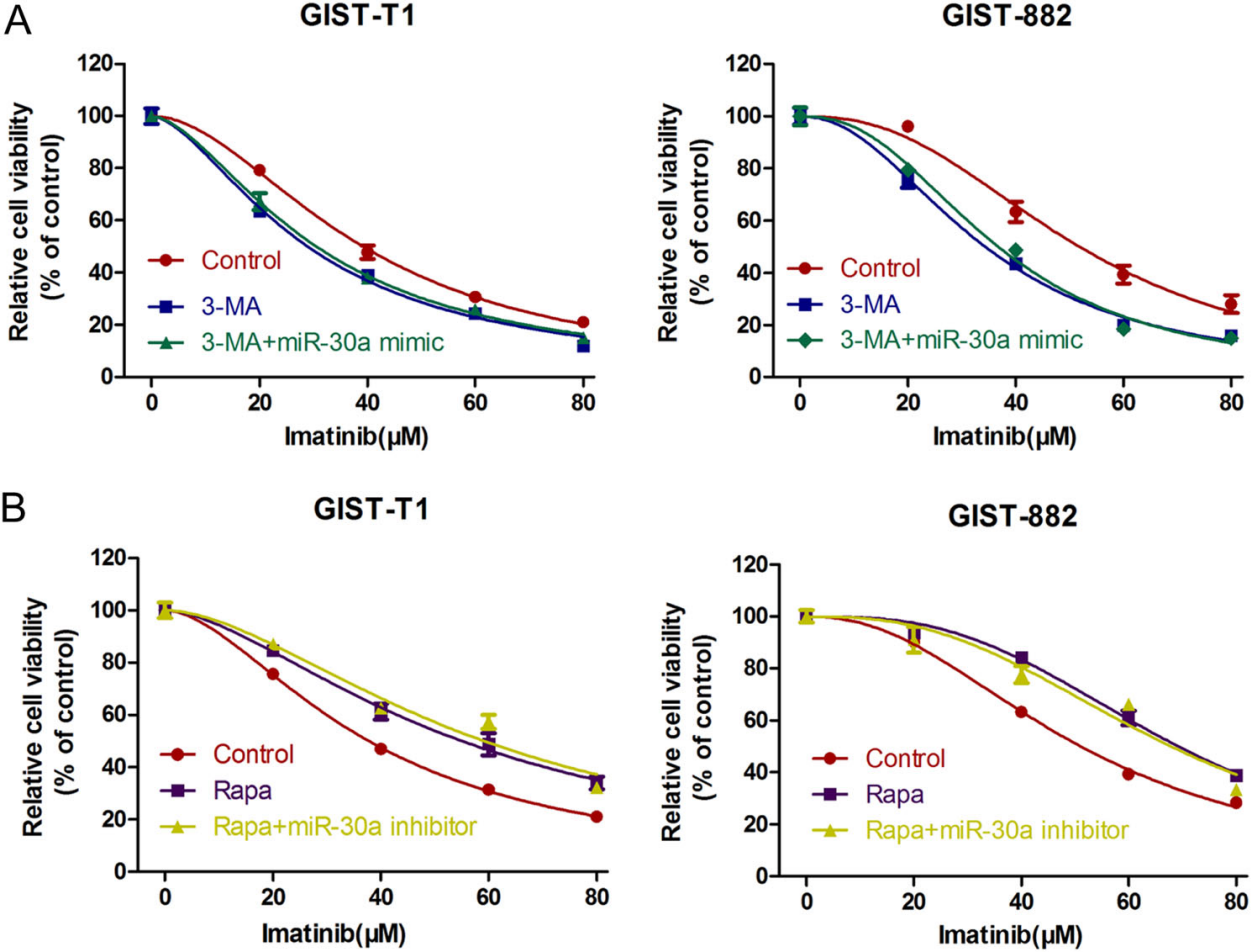
MiR-30a sensitizes GIST cells to imatinib via autophagy. **a** GIST-T1 and GIST-882 cells treated with single 3-MA, 3-MA, and miR-30a inhibitor or the negative control were exposed to imatinib, then the cell viability was measured by the CCK-8 assay. **b** GIST-T1 and GIST-882 cells were treated with Rapa, Rapa and miR-30a inhibitor or the negative control, and the cell viability was measured by CCK-8 assay in the presence of various concentrations of imatinib. All data are representative of three independent experiments.

To confirm that miR-30 increased cell sensitivity to IM by regulating cell autophagy, we used 3-MA, an autophagy inhibitor and an autophagy inducer (rapamycin, RAPA) to interfere in the autophagic process. Interestingly, in the presence of 3-MA, the miR-30 mimic no longer protected GIST cells against IM cytotoxicity (Fig. 5a). After treatment with RAPA, the miR-30 inhibitor also had no obvious influence on GIST cell viability in the context of IM intervention (Fig. 5b). Therefore, our findings confirmed that miR-30 increases cell sensitivity to IM by regulating cell autophagy.

### Beclin-1 is a target gene of miR-30

**Fig. 6:**
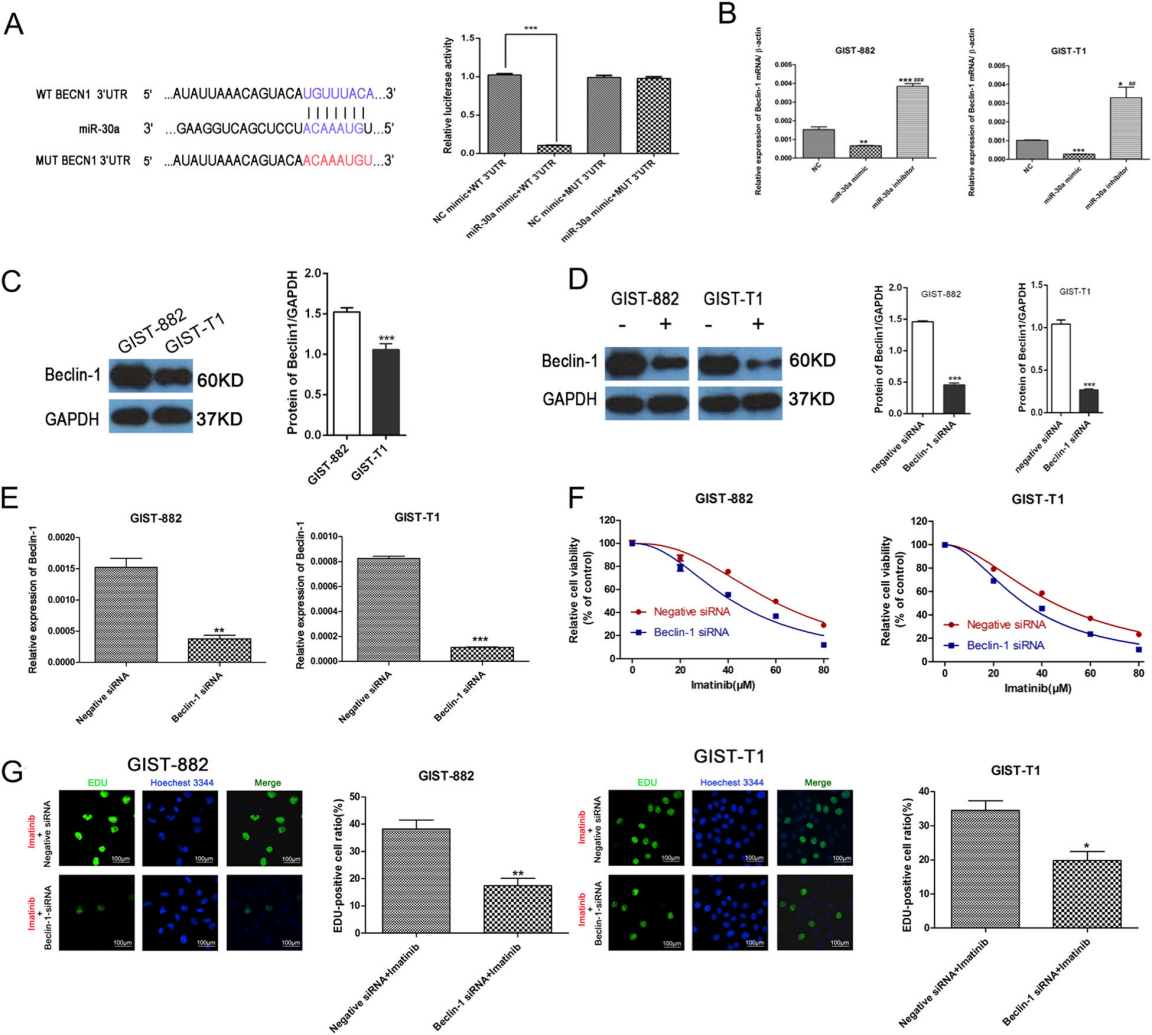
Beclin-1 is a target gene of miR-30a and Beclin-1 knockdown increases imatinib sensitivity in GIST cells. **a** Results of Luciferase reporter gene assays. ***P < 0.001. **b** qRT-PCR was performed to detect the relative beclin-1 expression level when miR-30a was upregulated and downregulated in GIST-T1 and GIST-882 cells. *P < 0.05, **P < 0.01, ***P < 0.001 vs. Negative Control; ^##^*P* < 0.01, ^###^*P* < 0.001 vs. miR-30a mimic. **c** The beclin-1 expression level in GIST cells was assayed by western blot. ***P < 0.001 vs. GIST 882 (**d**) knockdown of beclin-1 was validated by western blot. ***P < 0.001 vs. NC (**e**) the beclin-1 expression level was assayed by qRT-PCR. **P < 0.01, ***P < 0.001 vs. Negative siRNA. **f**, **g** In parallel, the GIST cell sensitivity to imatinib indicated by cell viability and proliferation was measured by CCK-8 and EdU assay, respectively. *P < 0.05, **P < 0.01 vs. Negative siRNA. All data are representative of three independent experiments.

Beclin-1, the first identified autophagy-related protein in cancer, has previously been identified as the direct target of miR-30a in various cancer types, including small cell lung cancer, pancreatic cancer, and cervical cancer, etc^20–25^. This was confirmed in our study using TargetScan (www.targetscan.org) and luciferase reporter assays (Fig. 6a). To confirm the existence of this interaction in GIST cells, we transfected GIST cell lines with miR-30a mimic, miR-30a inhibitor or negative control. Changes in beclin-1 expression under the influence of three different miR-30a levels were then analyzed by qRT-PCR. As shown in Fig. 6b, c, the relative level of beclin-1 was lower in the miR-30a mimic transfected group compared with those in the control group, while the miR-30a inhibitor effectively elevated beclin-1 expression levels. These findings suggested that beclin-1 is a target gene of miR-30 and that miR-30 negatively regulates beclin-1.

### Knockdown of beclin-1 increased drug sensitivity in GIST cell lines

Evaluation of the basal expression of beclin-1 in GIST-882 and GIST-T1 cell lines showed that higher beclin-1 levels in GIST-882 cells than those in GIST-T1 cells (Fig. 6c), which might be related to the higher tolerance of GIST-882 cells to IM treatment. Subsequently, we showed that siRNA-mediated knockdown of beclin-1 expression in GIST cells (Fig. 6d, e). This finding showed that beclin-1 was successfully silenced. To investigate the role of beclin-1 in IM resistance of GIST cells, we evaluated cell viability and proliferation after IM treatment in GIST-882 and GIST-T1 cells following beclin-1 knockdown. The results showed that beclin-1 knockdown significantly increased the chemosensitivity of the two GIST cell lines (Fig. 6f, g), indicating that similar to miR-30a upregulation, beclin-1 downregulation sensitized GIST cells to IM.

### MiR-30a increased imatinib sensitivity of GIST cell via beclin-1 downregulation

**Fig. 7:**
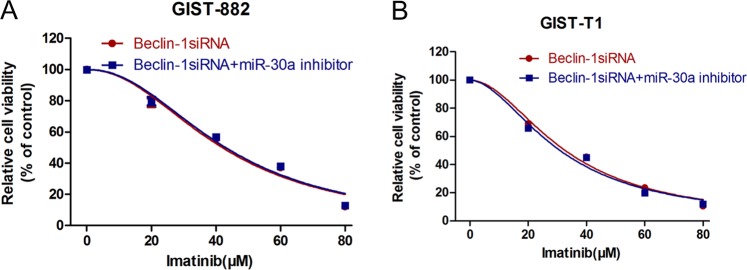
MiR-30a enhances imatinib sensitivity of GIST cells by downregulation of beclin-1. **a**, **b** Beclin-1 siRNA alone or the combination of beclin-1 siRNA and miR-30a inhibitor were transfected into the GIST cells, then the cell viability following imatinib treatment was measured by the CCK-8 assay. All data are representative of three independent experiments.

Given that beclin-1 is a downstream target of miR-30a and both beclin-1 and miR-30a are involved in chemosensitivity to IM, we hypothesized that miR-30a increases GIST cell sensitivity to IM via beclin-1. To test our hypothesis, we co-transfected GIST-882 and GIST-T1 cells with the miR-30a inhibitor and beclin-1 siRNA. Intriguingly, miR-30a knockdown in the context of siRNA-mediated beclin-1 inhibition displayed no significant difference in IM sensitivity compared to that of the cells transfected with beclin-1 siRNA alone (Fig. 7). This result suggested that miR-30a affects IM sensitivity of GIST cell through downregulation of beclin-1.

### MiR-30a enhanced imatinib sensitivity of GIST cells in vivo

**Fig. 8:**
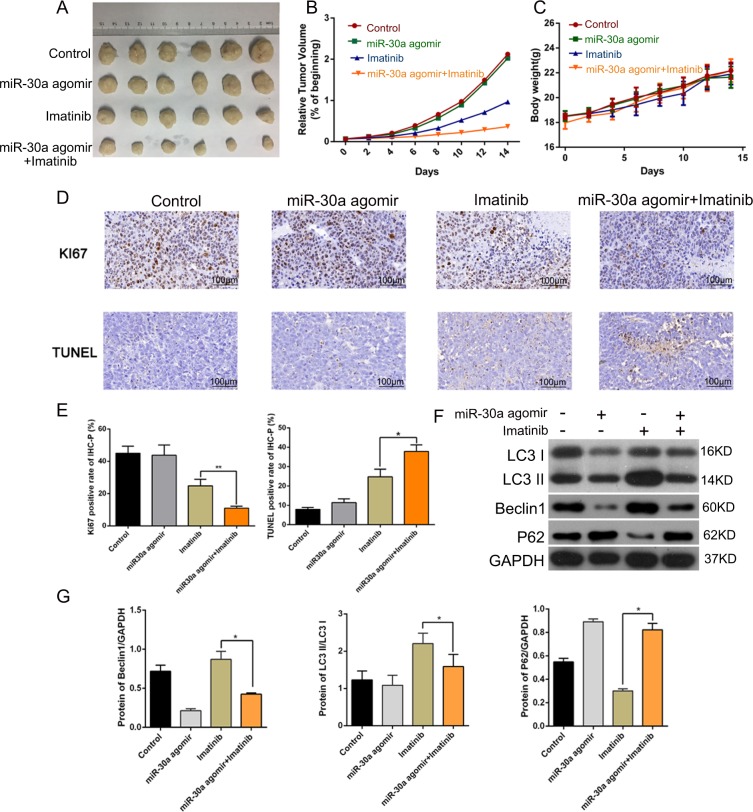
MiR-30a increases imatinib sensitivity of GIST cells in mouse models. **a** Tumor sizes in mice after 2 weeks of treatment with or without imatinib in the presence or absence of agomiR-30a. **b**, **c** During the 2 weeks, relative tumor volume and body weight were monitored. **d**, **e** Tumor cell proliferation was evaluated by Ki-67 immunohistochemical staining, and apoptosis by TUNEL assay. **f**, **g** The autophagy-related proteins were measured by Western blot. *P < 0.05, **P < 0.01 vs. IM. All data are representative of three independent experiments.

We further explored the ability of miR-30a to sensitize GIST cells to IM in vivo using a mouse tumor xenograft model. As shown in Fig. 8a, b, the application of agomiR-30a had no significant effect on tumor growth compared with the control, indicating that the agomiR-30a markedly increased IM toxicity. Administration of agomiR-30a and IM alone or in combination did not result in a noticeable change in the body weight of the mice, suggesting an absence of obvious toxicity of these treatments in the mice (Fig. 8c). In accordance with the effects on tumor volume, immunohistochemical staining showed that the lowest percentage of Ki67-positive proliferating cells in the miR-30a upregulated and IM treated group; the apoptosis rate detected by TUNEL assay was also highest in this group (Fig. 8d, e). The results of western blotting also confirmed that autophagy was enhanced by IM, and miR30 attenuated IM-induced autophagy in vivo (Fig. 8f, g). Collectively, these results indicated that miR-30a strengthened IM toxicity in GIST cells in vivo.

## Discussion

Gastrointestinal stromal tumors (GISTs), with a prevalence of approximately 130 cases per million of the population globally, is the most common category (approximately 20%) of all sarcomas^4^. Mutations in *KIT* or *PDGFRA* occur in 90% to 95% of GISTs, hence IM, a selective inhibitor targeting KIT, PDGFRs, and some other tyrosine kinases, was developed and shown to greatly benefit patients with advanced GISTs^4,8,9^. However, most patients who were primarily sensitive to IM acquired drug resistance within 2–3 years^9^. Although newly acquired mutations in *KIT* or *PDGFRA* that interfere with the IM-binding sites account for 70% to 80% of all cases of secondary resistance, alternative pathways were activated in more than 10% of patients^26^. For instance, it was reported that the activation of FGFR3 by FGF2 reduced the effectiveness of IM in IM-sensitive GIST cells, and elevated FGF2 levels were detected in IM-resistant clinical GIST samples^27^. Therefore, we investigated alternative pathways that might be responsible for KIT-independent IM resistance in GISTs.

MiR-30a participates in a range of biological processes in cancer, including cell proliferation, invasion, metastasis, and autophagy, and functions as either a proto-oncogene or a tumor suppressor^15^. Based on its multiple roles in tumorigenesis and progression, its involvement in chemotherapy resistance has been widely explored. Generally, miR-30a levels correlated negatively with resistance to anti-cancer drugs, whether they were either traditional chemotherapy drugs, such as cisplatin, or molecular targeted drugs^19,21,28–31^. The mechanisms underlying the increased drug resistance caused by downregulation of miR-30a varied from the crosstalk with apoptotic pathways, to activation of alternative pathways, to induction of autophagy^19,21,28–31^. However, a study involving CAGE (a cancer/testis antigen)-expressing hepatoma and melanoma cells showed that miR-30a decreased the expression of p53 in a CAGE-dependent manner, leading to resistance to a HER-2 inhibitor, trastuzumab^32^. Therefore, the role of miR-30a in chemotherapy drug resistance may vary in different contexts. In our study, miR-30a levels were shown to be lower in GIST-882 cells with relatively higher IM IC_50_ compared to GIST-T1 cells, and downregulation of the miR-30a conferred IM resistance to both cell lines, indicating that miR-30a serves as an IM sensitizer in GIST cells.

Autophagy is a cytoprotective process in IM-refractory GIST cells^18^, but the mechanism by which autophagy is initiated in this situation is not clear. Previous study in chronic myeloid leukemia cells has revealed that miR-30a participates in the regulation of autophagy, and miR-30a downregulation of reduces IM toxicity by activating autophagy^19^. Here, we provide further evidence in GIST cells that miR-30a suppresses autophagy during IM treatment and miR-30a sensitizes GIST cells to IM via an autophagy-related mechanism. Given that miR-30a depends on autophagy to regulate IM sensitivity, we further reveal that miR-30a affects autophagy via beclin-1, a key protein required for autophagosome formation^33^. Specifically, we confirm that in GIST cells, beclin-1 is a target of miR-30, which is consistent with reports of other cancer types^20–25^. In addition, beclin-1 knockdown has a similar effect to miR-30a in IM sensitivity, with miR-30a increases IM sensitivity of GIST cell through downregulation of beclin-1. Our findings provide compelling evidence for the mechanism underlying autophagy initiation in IM-resistant GIST cells. However, numerous oncogenes and tumor suppressor genes are involved in the regulation of autophagy^20^, and other possible mechanisms remain to be explored.

It is worth noting that inhibiting autophagy does not seem to work in all situations. For instance, autophagy contributes to EGFR inhibitor responses in non-small-cell lung carcinoma cells with active EGFR mutations, and therefore, inhibiting autophagy in patients treated with EGFR TKIs may not benefit their clinical outcomes^34^. In addition, animal studies revealed some side-effects of autophagy inhibitors, such as the increased possibility of neuropathy, secondary cancers, infections, or metabolic disturbances^35^. Nevertheless, combining autophagy inhibitors with first-line therapies has succeeded in overcoming resistance or increasing sensitivity to the front-line drugs in clinical hematopoietic disorders, including several types of leukemia^36^. Moreover, more than 40 clinical trials investigating treatment of various cancers with autophagy inhibitors were recently found on the website, ClinicalTrials.Gov, although clinical trials on GIST patients are not included. Based on our findings, miR-30a, is an upstream regulator of autophagy in GIST cell lines. Furthermore, our demonstration of the therapeutic benefits of the agomiR-30a in a mouse tumor xenograft model indicates the potential of this approach to provide a favorable effect on the clinical course of IM-resistant GIST patients.

Importantly, to overcome IM resistance in GIST cases, further studies are required. On one hand, though playing an important role, beclin-1 is not the only target of miR-30a regulation and ATG5/ATG12 may also contribute to the biological effects of miR-30a in GIST according to target prediction. It has been reported that Atg5 is the target of miR-30a in chronic myeloid leukemia cells^19^. On the other hand, the miR-30 family shares the same target spectrum. The other miR-30 family members may also contribute to regulating autophagy. To fully dissect the mechanics of autophagy regulation, further investigation needs to be performed on these two aspects.

## Materials and methods

### Cell lines

Human GIST cell lines (GIST-882, GIST-T1) were obtained from the BeNa Culture Collection (Beijing, China) and were cultured in Dulbecco’s modified Eagle’s medium (Gibco, Grand Island, NY, USA) supplemented with 10% fetal bovine serum (Gibco) and 1% penicillin/streptomycin (Sigma-Aldrich, St. Louis, MO). Cells were maintained at 37 °C in a humidified incubator under 5% CO_2_ and were used within 3 months of resuscitation.

### Real-time quantitative reverse transcription polymerase chain reaction

Total RNA was extracted from tissues using TRIzol (Invitrogen, CA, USA). Reverse transcription was performed with PrimeScript RT reagent Kit (Takara, Japan) according to the manufacturer’s protocol. Real-time quantitative reverse transcription polymerase chain reaction (qRT-PCR) was performed using SYBR Green (TaKaRa Biotechnology, Dalian, China) on an ABI Prism 7900HT Real-Time System (Applied Biosystems Inc., Foster City, CA, USA). RT-PCR was performed with the following cycling conditions: 95 °C for 30 s, 40 cycles of 95 °C for 5 s, and 60 °C for 30 s. The expression levels of miRNAs in each group were calculated by relative quantification (2^-ΔΔCt^), which was normalized to U6 rRNA. All assays were performed in triplicate. The miR-30 family primers were synthesized by Gemma (Shanghai, China). Other primers were synthesized by Takara. Primer sequences are listed in Supplementary Table 1.

### Cell viability and proliferation assays

To evaluate relative cell viability, GIST cells (5000 per well) were seeded into 96-well microplates and incubated overnight. The culture medium was then replaced with complete media containing IM (0, 20, 40, 60, and 80 µmol/L) for 48 h. Cell viability was determined using then the cell counting kit-8 assay (CCK-8; KeyGEN) according to the manufacturer’s instructions. Cell viability was expressed relative to that of the untreated control cells.

To evaluate cell proliferation, cells were treated with IM at the appropriate half maximal inhibitory concentration (IC50). The cells were then assayed using the Click-iT 5-ethynyl-20-deoxyuridine (EdU) Imaging Kit (Invitrogen, Carlsbad, CA, USA) following the manufacturer’s instructions and counterstained with Hoechst 33342. The percentage of proliferating cells in five random fields of view per slide was determined under an inverted fluorescence microscope (Olympus, Tokyo, Japan) and expressed relative to that in the untreated control cells.

### Apoptosis

GIST cells or transfected cells were treated with IM (IC50) for 48 h, stained using the Annexin V-PE/7AAD apoptosis kit (BD Biosciences) according to the manufacturer’s protocol. Data were acquired using a BD FACS Caliber flow cytometer and analyzed using BD Cell Quest software.

### Cell transfection

Asynchronously growing cells were seeded in 6-well plates at 2 × 10^5^ cells/well. Hsa-miRNA-30a mimic, hsa-miRNA-30a inhibitor, and nonspecific control were purchased from RiboBio (Guangzhou, China). Transfections were performed using the Lipofectamine 2000 Kit (Invitrogen) according to the manufacturer’s instructions.

### Dual luciferase reporter assay

The 3’-untranslated region (UTR) and mutated 3’-UTR of the amplified BECN1 fragment was cloned into the pGL3 vector containing the firefly luciferase reporter gene (Promega, Madison, WI, USA). Subsequently, 293 T cells were cotransfected with 200 ng of firefly luciferase construct, 4 ng of pRL-TK Renilla luciferase plasmid and 50 nM of miR-30a mimic according to the manufacturer’s protocol supplied with the luciferase reporter assay kit (Promega, Madison, WI). At 48 h after transfection, relative Renilla luciferase activity (firefly luciferase/Renilla luciferase) was measured in dual luciferase reporter assays.

### Western blot analysis

Total cell proteins were isolated using cell lysis buffer. Equal amounts of protein (40 µg) were separated by 10% sodium dodecyl sulfate-polyacrylamide gel electrophoresis and then transferred to polyvinylidene difluoride membranes (Millipore, Bedford, MA, USA) by electroblotting. The membranes were blocked with 5% bovine serum albumin, and then incubated with the appropriate primary detection antibody (all at 1:1000 dilution) overnight at 4 °C. Membranes were then incubated with horseradish peroxidase (HRP)-conjugated secondary antibodies (all at 1:2000 dilution). All antibodies were purchased from Cell Signaling Technology except the HRP-conjugated secondary antibodies (Beyotime Institute of Biotechnology).

### mRFP-GFP-LC3 analysis

Cells transfected with a mRFP-GFP-LC3 adenovirus or a control were treated with or without IM. After 24 h, cells were fixed in 3.7% formaldehyde for 20 min at room temperature, washed three times with phosphate-buffered saline (PBS), mounted on glass slides and examined using laser scanning confocal microscope (LSM 800). Autophagosomes were identified in confocal images by merging the red and green fluorescent regions merge. The red spots indicated autophagic lysosomes. Differences in the color intensity indicate the strength of autophagy flow.

### Transmission electron microscopy

Cells were seeded in 24-well plates and then fixed with fixative buffer containing 3% glutaraldehyde and 2% paraformaldehyde in 0.1 M PBS. After dehydration, samples were cut into sections (0.12 μm thickness) and stained with 0.2% lead citrate and 1% uranyl acetate. Digital images were obtained using the EM 1011CX electron microscope (JEOL, USA, Inc.) imaging system.

### Tumor xenograft studies

All animal experiments were performed in accordance with the Guide for the Care and Use of the Animal Ethics Committee of Zhejiang University (Hangzhou, China).

Male nude mice (aged 3–4 weeks, 16–20 g; Silaike Experimental Animal Center; Shanghai, China) were housed under pathogen-free conditions and supplied with irradiated feed. Mice were divided randomly into four groups (*n* = 6/group) and then injected subcutaneously in the right axillary fossa with approximately 1 × 10^6^ GIST-882 cells in 100 μl PBS. Tumor volumes were estimated on alternate days according to the following formula: volume = (tumor length × tumor width^2^)/2. When the tumor volume reached 50–100 mm^3^, treatment was initiated. Mice received IM (100 mg/kg), or vehicle (control group; equal volume of diluents) intraperitoneally, and/or agomiR-30a (2 nmol) intratumorally every 2 days. After 2 weeks of treatment, mice were sacrificed and tumors were dissected out and measured. Tumor cell proliferation and apoptosis were evaluated by Ki-67 immunohistochemical staining and TUNEL assays, respectively.

### Statistical analysis

All the experiments were performed in triplicate. Quantitative values were expressed as the mean ± standard error of the mean (SEM). Statistical analysis was performed by one-way ANOVA using GraphPad Prism software (San Diego, CA, USA); and *P* < 0.05 was considered to indicate statistical significance.

## Supplementary information

supplementary figure legends

Figure S1

supplementary table legends

supplementary Table1

Declaration of contrubution to artical
