# Supplementary material for: MicroRNA-30a targets BECLIN-1 to inactivate autophagy and sensitizes gastrointestinal stromal tumor cells to imatinib
Source: Cell Death Dis. 2020 Mar 23;11(3):198. doi: 10.1038/s41419-020-2390-7 (PMC7090062; doi:10.1038/s41419-020-2390-7)
Supplement: Supplementary file 1 — supplementary figure legends [file 41419_2020_2390_MOESM1_ESM.docx]

**Supplementary figure legends**

**Figure S1 Comparison of mir-30a expression level in** **gastrointestinal stromal tumor and cell lines.**

(A) qRT-PCR was used to assess a series of miRNA levels in GIST-882 cells treated with IM.*p<0.05, **p<0.01. (B) qRT-PCR was used to assess a series of miRNA levels in GIST-T1 cells treated with IM.*p<0.05,**p<0.01. (C) Comparison of mir-30a expression in gastrointestinal stromal tumor tissues compared to normal adjacent normal tissues. *p<0.05, **p<0.01. All data are representative of three independent experiments.

**Supplementary table legends**

Table1. All the primer sequences were showed as followed.
