# Supplementary figures and images for: MicroRNA-30a targets BECLIN-1 to inactivate autophagy and sensitizes gastrointestinal stromal tumor cells to imatinib
Source: Cell Death Dis. 2020 Mar 23;11(3):198. doi: 10.1038/s41419-020-2390-7 (PMC7090062; doi:10.1038/s41419-020-2390-7)

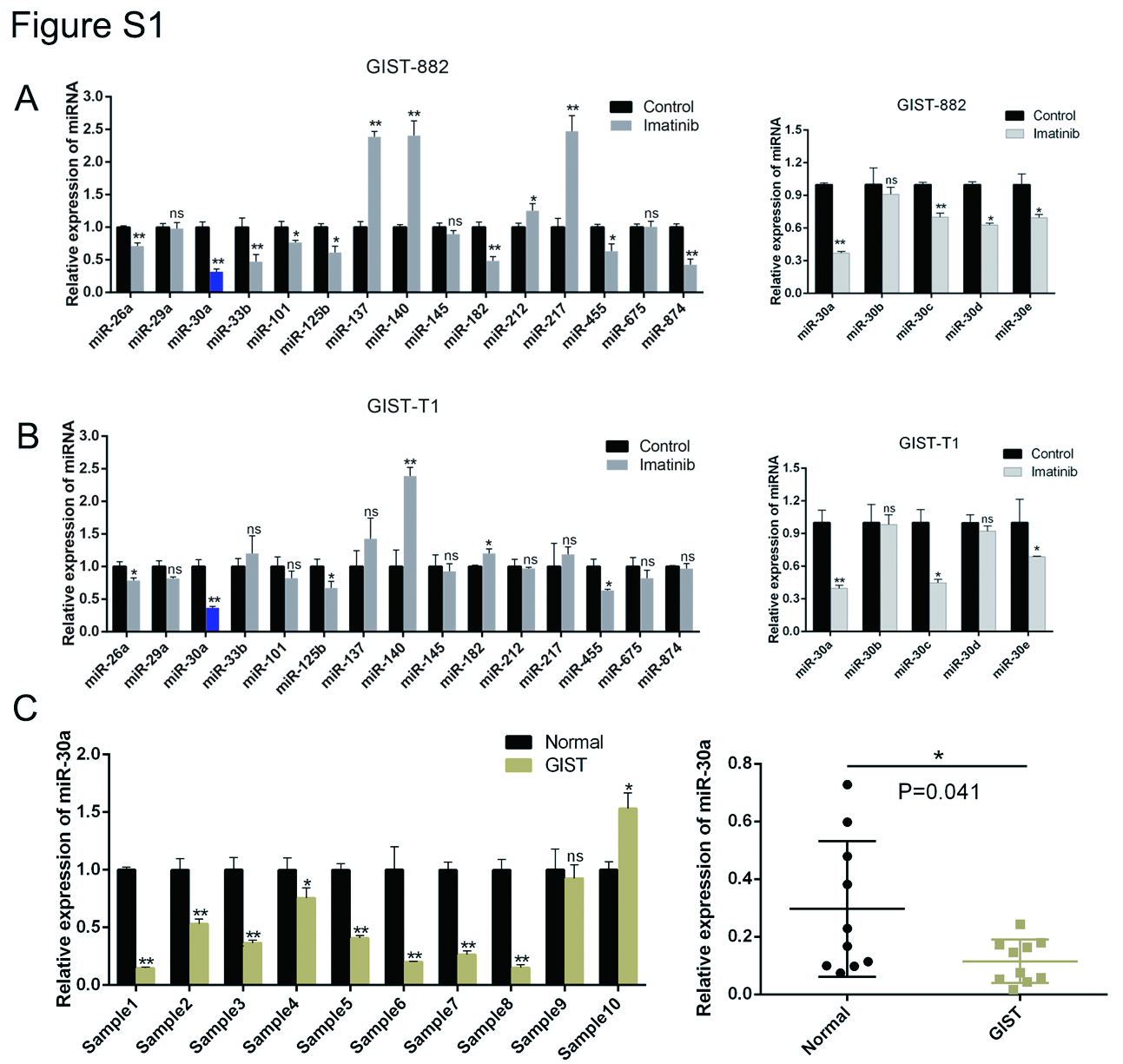

Supplement: Supplementary file 2 — Figure S1 [file 41419_2020_2390_MOESM2_ESM.tif]
