# Supplementary material for: MicroRNA-30a targets BECLIN-1 to inactivate autophagy and sensitizes gastrointestinal stromal tumor cells to imatinib
Source: Cell Death Dis. 2020 Mar 23;11(3):198. doi: 10.1038/s41419-020-2390-7 (PMC7090062; doi:10.1038/s41419-020-2390-7)
Supplement: Supplementary file 3 — supplementary table legends [file 41419_2020_2390_MOESM3_ESM.docx]

**Supplementary table legends**

Table1. All the primer sequences were showed as followed.
