# Supplementary material for: MicroRNA-30a targets BECLIN-1 to inactivate autophagy and sensitizes gastrointestinal stromal tumor cells to imatinib
Source: Cell Death Dis. 2020 Mar 23;11(3):198. doi: 10.1038/s41419-020-2390-7 (PMC7090062; doi:10.1038/s41419-020-2390-7)
Supplement: Supplementary file 4 — supplementary Table1 [file 41419_2020_2390_MOESM4_ESM.docx]

|  | | 5’- 3’ |
| --- | --- | --- |
| Has-miR-30a F | CACTCTCATGTAAACATCCTCGAC | |
| Has-miR-30a R | TATGGTTTTGACGACTGTGTGAT | |
| Has-miR-30b F | GCCGCTGTAAACATCCTACAC | |
| Has-miR-30b R | TATGGTTTTGACGACTGTGTGAT | |
| Has-miR-30c F | CAACCAATGTAAACATCCTACACTC | |
| Has-miR-30c R | TATGGTTTTGACGACTGTGTGAT | |
| Has-miR-30d F | AATCTTTGTAAACATCCCCGACT | |
| Has-miR-30d R | TATGCTTGTTCTCGTCTCTGTGTC | |
| Has-miR-30e F | GATTCATTCGTGTAAACATCCTTG | |
| Has-miR-30e R | TATGCTTGTTCTCGTCTCTGTGTC | |
| hsa-miR-26a | TTCAAGUAATCCAGGATAGGCT | |
| hsa-miR-29a | ACTGATTTCTTTTGGTGTTCAG | |
| hsa-miR-33b | GTGCATTGCTGTTGCATTGC | |
| hsa-miR-101 | CAGTTATCACAGTGCTGATGCT | |
| hsa-miR-125b | TCCCTGAGACCCTAACTTGTGA | |
| hsa-miR-137 | ACGGGTATTCTTGGGTGGATAAT | |
| hsa-miR-140 | CAGTGGTTTTACCCTATGGTAG | |
| hsa-miR-145 | GTCCAGTTTTCCCAGGAATCCCT | |
| hsa-miR-182 | TTTGGCAATGGTAGAACTCACACT | |
| hsa-miR-212 | ACCTTGGCTCTAGACTGCTTACT | |
| hsa-miR-217 | TACTGCATCAGGAACTGATTGGA | |
| hsa-miR-455 | TATGTGCCTTTGGACTACATCG | |
| hsa-miR-675 | TGGTGCGGAGAGGGCCCACAGTG | |
| hsa-miR-874 | CGGCCCCACGCACCAGGGTAAGA | |
