# Supplementary material for: MicroRNA-30a targets BECLIN-1 to inactivate autophagy and sensitizes gastrointestinal stromal tumor cells to imatinib
Source: Cell Death Dis. 2020 Mar 23;11(3):198. doi: 10.1038/s41419-020-2390-7 (PMC7090062; doi:10.1038/s41419-020-2390-7)
Supplement: Supplementary file 5 — Declaration of contrubution to artical [file 41419_2020_2390_MOESM5_ESM.pdf]

DECLARATION OF CONTRIBUTIONS TO ARTICLE

ADMC

Manuscript Number: CDDIS-19-1472RRRRR

Journal Name: *Cell Death & Disease* (the 'Journal')

Proposed Title of the Contribution:

MicroRNA-30a targets BECLIN-1 to inactivate autophagy and sensitize gastrointestinal stromal tumor cells to imatinib (the 'Contribution)

Author(s):

Wei Chen, Zhouqi Li,Hao Liu, Sujing Jiang, Guannan Wang, Lifeng Sun, Jun Li, Xiaochen Wang, Shaojun Yu, Jianjin Huang, Ying Dong (the 'Authors')

For all *CDDis* articles, each person named as an author in the published version must be able to show he or she has contributed substantially to the article.

Authorship credit should be based on 1) substantial contributions to conception and design, acquisition of data, or analysis and interpretation of data; 2) drafting the article or revising it critically for important intellectual content; and 3) final approval of the version to be published. Authors should meet conditions 1, 2 and 3.

Any person who cannot be shown to have made a substantial contribution to the article cannot be listed as an author in the final version. The name of any person who is deemed to have made a minor contribution can, however, appear in the Acknowledgments section of the article.

Please complete the table below to indicate the contributions of all named authors to the manuscript.

Author Full Name:	Specification of Contribution to the Manuscript:
Wei Chen	1,wrote the paper;2,confirmation of the final manuscript;3,submission of the papers
Zhouqi Li	1,re-wrote the paper;2,performed the experiments;3,confirmation of the final manuscript;
Hao Liu	1,re-wrote the paper;2,confirmation of the final manuscript;3,performed the experiments
Sujing Jiang	1,performed the experiments;2,confirmation of the final manuscript;
Guannan Wang	1,performed the experiments;2,confirmation of the final manuscript;
Lifeng Sun	1,participated in the data collection and analysis;2,confirmation of the final manuscript;
Jun Li	1,participated in the data collection and analysis;2,confirmation of the final manuscript;
Xiaochen Wang	1,participated in the data collection and analysis;2,confirmation of the final manuscript;
Shaojun Yu	1,participated in the data collection and analysis;2,confirmation of the final manuscript;
Jianjin Huang	1,participated in the data collection and analysis;2,confirmation of the final manuscript;
Ying Dong	1,conceived and designed the research;2,reviewed and revised the manuscript;3,confirmation of the final manuscript;

Please complete the table below to indicate the contributions of all named authors to the figures.

Figure 1:

Zhouqi Li and Sujing Jiang performed the experiment. Guannan Wang, Lifeng Sun, Jun Li, Xiaochen Wang, Shaojun Yu and Jianjin Huang collected and analyzed the data.

Figure 2:

Zhouqi Li and Sujing Jiang performed the experiment. Guannan Wang, Lifeng Sun, Jun Li, Xiaochen Wang, Shaojun Yu and Jianjin Huang collected and analyzed the data.

Figure 3:

Zhouqi Li and Sujing Jiang performed the experiment. Guannan Wang, Lifeng Sun, Jun Li, Xiaochen Wang, Shaojun Yu and Jianjin Huang collected and analyzed the data.

Figure 4:

Zhouqi Li and Sujing Jiang performed the experiment. Guannan Wang, Lifeng Sun, Jun Li, Xiaochen Wang, Shaojun Yu and Jianjin Huang collected and analyzed the data.

Figure 5:

Zhouqi Li and Sujing Jiang performed the experiment. Guannan Wang, Lifeng Sun, Jun Li, Xiaochen Wang, Shaojun Yu and Jianjin Huang collected and analyzed the data.

Figure 6:

Zhouqi Li and Sujing Jiang performed the experiment. Guannan Wang, Lifeng Sun, Jun Li, Xiaochen Wang, Shaojun Yu and Jianjin Huang collected and analyzed the data.

Signed for and on behalf of the Author(s):	Print Name:	Date:
Ying Dong	Ying Dong	2020-2-22
